# Sharing Channel Strategy With Customers’ Collaborative Consumption Behaviors

**DOI:** 10.3389/fpsyg.2022.792704

**Published:** 2022-03-28

**Authors:** Shaozeng Dong, Mingqiao Luan, Lingming Chen, Zulqurnain Ali

**Affiliations:** ^1^School of Business, Jiangsu Ocean University, Lianyungang, China; ^2^Executive Training College of China Energy, Beijing, China; ^3^School of Economics and Management, Xinyu University, Jiangxi, China; ^4^IRC for Finance and Digital Economy, KFUPM Business School, King Fahd University of Petroleum and Minerals, Dhahran, Saudi Arabia

**Keywords:** collaborative consumption, pricing, sharing economy, sharing channel, customer behavior

## Abstract

Associated with the sharing economy, collaborative consumption behaviors often take place among customers. Different from the traditional consumption that customers purchase the product and own it, in the sharing economy, customers can access the product only for a particular period and the ownership of the product also belongs to the firm. In this paper, we develop a theoretical analysis model, and investigate the intrinsic connection between collaborative consumption and the sharing channel strategy. Adopting the sharing channel strategy, the firm has a chance to expand the market demand and improve its profit. In addition, we examine the impacts of other influential factors on a firm’s decisions, such as the unit product cost, surplus-value, and service capability coefficient.

## Introduction

In this age of globalization, collaborative consumption has sprung up, that is closely associated with the sharing economy (Schwanholz and Leipold, 2020). Collaborative consumption is a subset of the overarching sharing economy. In a traditional economy, a used-goods sale transaction involves a permanent transfer of product ownership from firms to buyers, in other words, buyers obtain all the rights of the product (Huber, 2017; Fraanje and Spaargaren, 2018). Whereas a product-sharing transaction involves merely a temporary transfer of the right to use a product from the firm to the buyer without transferring the product ownership (Belk, 2014; Jiang and Tian, 2016). Besides obtaining the right to use a product from firms, such as the operation forms of ofo, Gofun, Uber, etc., a product user can also obtain the right to use a product from other users, such as the operation forms of Anbribn, NeighborGoods, etc. (Ertz et al., 2018). As collaborative consumption affects not only customers’ purchase decisions but also firms’ distribution channels strategy, the debate regarding the future of consumption has become a focal subject both in practice and academia (Lindblom et al., 2018; Park and Armstrong, 2019).

Customers’ consumption paradigm has changed a lot with the development of sharing economy. In many cases, customers only expect to obtain the right to use a product for a certain period of time with no expectations of owning it. In the framework of sharing economy, such sharing activities of customers refer to collaborative consumption. Examples of collaborative consumption are renting, lending, bartering, reselling, trading, swapping, etc. (Möhlmann, 2015).

Collaborative consumption may influence firms’ distribution channels strategies a lot, such as introducing sharing channels to their traditional distribution strategies. Sharing channels are beneficial to firms. On the one hand, a sharing channel has a cannibalization effect on the traditional channel, offering customers an alternative to share the product while lowering the demand intercept in the traditional channel (Tian and Jiang, 2017). On the other hand, a sharing channel not only keeps concurrent customers who are familiar with collaborative consumption, but also attracts potential customers to get access to the product without owning it.

Based on the above analysis, we come up with the following research questions. How does the sharing channel affect customers’ purchase decisions? How does collaborative consumption affect firms’ distribution channels strategies? In addition, what is the intrinsic connection between the sharing channel and collaborative consumption? In this paper, we address the aforementioned questions by developing an analytical framework, in which a firm chooses to introduce the sharing channel or not, to investigate customers’ collaborative consumption behaviors.

The rest of our paper is organized as follows. In section “Literature Review,” we review the related literature. In section “Methodology and data,” we present our model. In section “Results,” we discuss the benchmark case in which the firm does not adopt the sharing channel strategy and the sharing channel strategy case where customers can select to purchase the product from the traditional channel or share the product from the sharing channel. We make a comparative analysis in section “Discussion and Conclusion.” And we conclude the paper with some discussions and directions for future research in section 6. All proofs of the propositions and lemmas are given in the Supplementary Appendix.

## Literature Review

The fast-growing trend in the sharing economy and collaborative consumption has attracted much attention in both practice and academia. Our paper focuses on emerging research literature on related topics. As product-sharing affects not only firms’ decisions, but also the whole distribution channels (Tian and Jiang, 2017), we will review the related references from both customers’ and firms’ perspectives.

First, from customers’ perspective, the sharing economy has changed customers’ consumption paradigm. With collaborative consumption, customers can get access to the product rather than owning it (Hartl et al., 2015). Tussyadiah (2015) explored factors that activate or deactivate collaborative consumption in the tourism market, concluding that driving factors of collaborative consumption include the societal aspects of sustainability, community, and economic benefits while hindering factors include lack of trust, lack of efficacy with regards to technology and lack of economic benefits. Benjaafar et al. (2019) analyze the impacts of collaborative consumption by describing an equilibrium model, and find that collaborative consumption is always beneficial to customers. Furthermore, Liu et al. (2016) propose a comprehensive theoretical model for customer information sharing behavior and find that customer information sharing is affected by both individual and social capital feedback. Besides, customers’ collaborative consumption behaviors are driven by perceived economic and environmental benefits, but not social benefits (Barnes and Mattsson, 2017). In addition, the service level will affect the ownership and usage of products (Agrawal and Bellos, 2017), which further affect customers’ collaborative consumption decisions. De et al. (2017) reveal the impacts of quality of service based pricing schemes for content sharing in peer-to-peer networks with a game-theoretic model, and the result shows that a higher upload capacity can foster rational sharing to start when the network is small, but discourages sharing behaviors when the network becomes large. Using a structural equation model, Hwang and Griffiths (2017) find that specific dimensions of value perceptions (utilitarian, hedonic and symbolic) have different effects on millennials’ attitudes and empathy toward collaborative consumption services.

Second, from firms’ perspective, collaborative consumption plays an important role in firms’ decisions as well as their distribution channels strategies. For instance, capacity sharing between competitors can solve the mismatch between supply and demand, which in turn impacts firms’ profitability and competition (Guo and Wu, 2018). Jiang and Tian (2016) investigate the impacts of customer-to-customer sharing of products in a monopolist market and find that the manufacture’s unit product cost and the product-sharing transaction cost determine the prosperity of sharing market. Li (2018) explores the impacts of cooperative purchasing and proactive inventory sharing on channel balancing, finding that these two strategies can lower the firms’ effective sourcing cost but are complementary in uncertain markets. In addition, researchers have investigated the utility of collaborative consumption in different industries, such as food delivery services (Correa et al., 2018), auto firms (Bellos et al., 2017), and fast fashion (Zamani et al., 2017), finding that collaborative consumption will strongly affect firms’ operation management strategies.

However, most of the aforementioned papers are empirical or experimental studies. Different from these papers, we establish an analytical framework to examine the impacts of customers’ collaborative consumption behaviors. Furthermore, as we stated earlier, most existing research on collaborative consumption and the sharing economy consider the impact of collaborative consumption on the firms’ performance, with few taking customers’ benefits into account, in our paper, we consider the intrinsic connection between customers’ collaborative consumption behaviors and the firm’s distribution channels strategy. In the following sections, we will analyze the equilibrium condition of adopting the sharing channel strategy and examine how the firm’s optimal sharing channel strategy is affected by a few influential factors.

## Methodology and Data

### Model Description

In this paper, we consider a monopoly market where a firm produces a product at the unit cost of *c* (0 < *c* < 1). Customers in the market with collaborative consumption behaviors are described as follows: one purchases the product and owns it, the other accesses to the product and only gets the right to use the product for a partial period. Customer i’s perceived value for the product follows uniform distribution from 0 to 1, and the total market demand is normalized to 1. To fit customers’ collaborative consumption behaviors, the firm decides to implement different distribution channel strategies: introducing the sharing channel or not. The firm’s effort to introduce the sharing channel is called “sharing channel” strategy throughout this paper. The underlining meaning is that customers are strategic, buying and owning the product from the traditional channel or only accessing the product from the sharing channel.

Sharing channel strategy has different impacts on the firm and customers. Form the firm’s perspective, sharing channel strategy helps establish an alternative product distribution channel between firms and customers. Since more product distribution channels will be more suitable for customers’ collaborative consumption behaviors, the sharing channel strategy may expand the market demand and earn higher profit for the firm. From the customers’ perspective, the sharing channel strategy supplies more alternative access to the product. In this way, there will be more customers sharing the product in the sharing channel. In other words, customers may choose only to get the right to use the product for a particular period, but give up the ownership of the product.

We assume all the customers make consumption decisions based on their individual utilities. If a customer can get more non-negative utilities from the traditional channel, he/she will choose to purchase and own the product. If a customer can get more non-negative utilities from the sharing channel, he/she will choose to access the product from the sharing channel.

**FIGURE 1:**
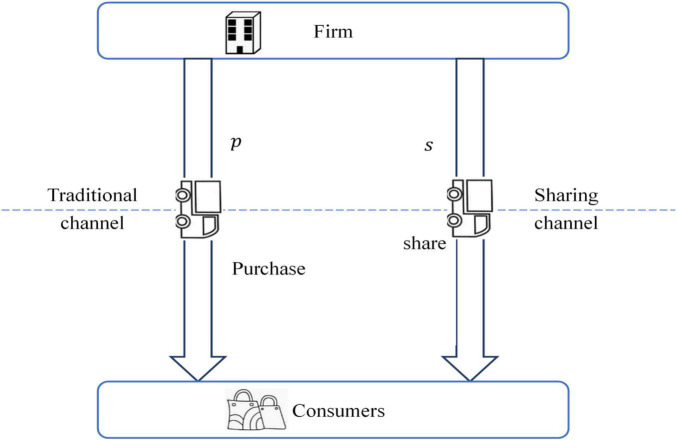
Sequential events.

The sequential events are plotted in Figure 1. At first, the firm decides the unit product price *p* in the traditional channel. If adopting the sharing channel strategy, the firm should announce the unit product sharing price *s* in the sharing channel. Also, introducing a sharing channel will increase the firm’s inputs, such as more staffs, more equipment, etc. Define $f=\frac{1}{2}\,k\,d_{s}^{2}$, which represents the firm’s inputs in introducing the sharing channel, where *d*_*s*_ is the market demand in the sharing channel, *k* is the channel service capability coefficient. High *k* means low channel service efficiency of the firm.

Customers with collaborative consumption behaviors should make strategic selections between the traditional channel and the sharing channel. If a customer selects to purchase the product in the traditional channel, he/she will take ownership of the product. We assume that there will be a δ surplus-value after the use of the product. To summarize, if a customer selects the traditional channel, he/she will get utility as *u*_*t*_ = *v*_*i*_ + δ−*p*. If a customer selects the sharing channel, his/her utility can be formulated as *u*_*s*_ = *v*_*i*_−*s*. When a customer’s utility satisfies the conditions of $\{\begin{array}{cc} u_{t} & \geq \\ u_{t} & \geq \end{array}\begin{array}{c} 0\\ u_{s} \end{array}$, the customer will select the traditional channel, buying and taking ownership of the product. When a customer’s utility satisfies the conditions of $\{\begin{array}{cc} u_{s} & \geq \\ u_{t} & < \end{array}\begin{array}{c} 0\\ u_{s} \end{array}$, the customer will select the sharing channel, only getting the right to use the product. In the sharing channel, the ownership of the product always belongs to the firm. Denote by *d* as the market demand, and we use subscript “*t*” or “*s*” to represent the variables in the traditional channel or the sharing channel, respectively.

We first consider a benchmark case where the firm does not adopt the sharing channel strategy. Then we consider a sharing channel case where the firm adopts the sharing channel strategy. We use superscript “*B*” and “*S*” to represent the benchmark case and the sharing channel case.

### Benchmark Case Without Sharing Channel Strategy

**FIGURE 2:**
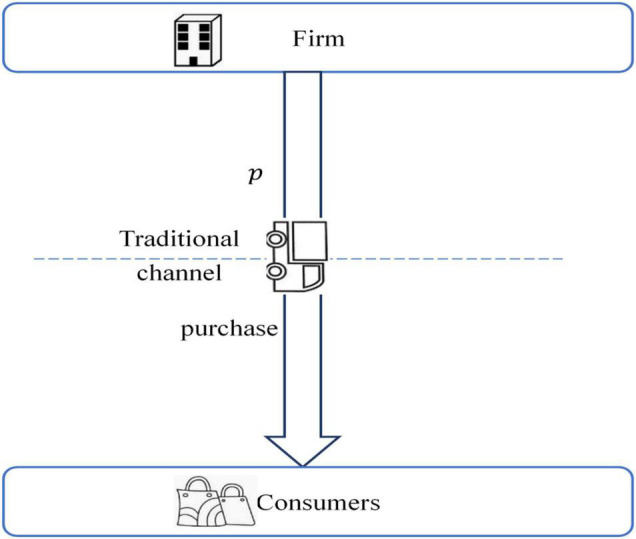
The sequential events of the benchmark case.

In this case, we consider that the firm does not adopt the sharing channel strategy. There is only one traditional distribution channel between the firm and customers. The sequential events of the benchmark case are shown in Figure 2.

The firm sells products to customers at product price $p_{t}^{B}$ through the traditional channel. Customers make their purchase decisions according to the product price and their individual utility. A customer will buy the product in the traditional channel if his/her utility is non-negative, i.e., $u_{t}=v_{i}+\delta -p_{t}^{B}\geq 0$; otherwise, he/she will not purchase the product. By solving the indifference utility between buying and not buying, we can derive the market demand of the traditional channel in the benchmark case as $d_{t}^{B}=1+\delta -p_{t}^{B}$. The firm’s profit maximize problem in the benchmark case can be formulated as below:


(1) $$\text{Max}\,\pi^{B}=\left(p_{t}^{B}-c\right)\,d_{t}^{B}$$

$$S.t.c<p_{t}^{B}<1$$

By solving formula (1), we can easily derive the firm’s optimal strategies in the benchmark case as shown in Proposition 1.

#### Proposition 1

In the benchmark case, the firm’s optimal pricing and corresponding solutions are as below:


$$p_{t}^{B^{*}}=\frac{1}{2}\left(1+c+\delta \right),d_{t}^{B^{*}}=\frac{1}{2}\left(1-c+\delta \right)and$$

$$\pi^{B^{*}}=\frac{1}{4}\,{\left(1-c+\delta \right)}^{2}.$$

### Case With Sharing Channel Strategy

In this case, we consider that the firm adopts the sharing channel strategy. Besides the traditional channel, the firm introduces a sharing channel to customers. The firm announces the product price $p_{t}^{S}$ in the traditional channel and simultaneously the unit product sharing price *s* in the sharing channel. Knowing the prices in the two channels, customers make channel selection decisions based on their individual utilities.

According to the model description in section “Methodology and data,” we see customers who select the traditional channel should satisfy the conditions as $\left\{ \begin{array}{c} u_{s}^{S}\geq 0\\ u_{t}^{S}\geq u_{s}^{S} \end{array}\right.$. On the other hand, when customers’ individual utilities satisfy the conditions of $\left\{ \begin{array}{c} u_{s}^{S}\geq 0\\ u_{t}^{S}<u_{s}^{S} \end{array}\right.$, they will select the sharing channel, obtaining the right to use the product but giving up the ownership. By solving the indifference utility between selecting the traditional channel and the sharing channel, we can derive the market demand of the traditional channel as $d_{t}^{S}=2\,\delta +s-p_{t}^{S}$ and the market demand of the sharing channel as $d_{s}^{S}=1-\left(\delta +s\right)$. The firm’s profit maximize problem in the sharing channel case can be formulated as below:


(2) $$\mathrm{Max}\,\pi^{S}=\left(p_{t}^{S}-c\right)\,d_{t}^{S}+\left(s+\delta -c\right)\,d_{s}^{S}-\frac{1}{2}\,k\,{\left(d_{s}^{S}\right)}^{2}$$

$$S.t.\left\{ \begin{array}{c} c<p_{t}^{S}<2\,\delta +s\\ m\,a\,x\,\left\{0,c-\delta \right\}<s<1-\delta \end{array}\right.$$

Formula (2) contains three parts: the first part is the firm’s net profit obtaining from the traditional channel, the second part is the firm’s profit obtaining from the sharing channel, and the last part is the firm’s input in introducing the sharing channel. The conditions of formula (2) ensure there always exists the traditional distribution channel and the sharing channel at the same time.

By solving the optimization problem of formula (2), we can characterize the firm’s optimal strategies under the sharing channel strategy, as presented in Proposition 2.

#### Proposition 2

Adopting the sharing channel strategy, the firm’s optimal pricing and corresponding solutions are shown as below:


$$p_{t}^{S^{*}}=\frac{1+k+c\,\left(2+k\right)+2\,\delta +k\,\delta }{3+2\,k};$$

$$s^{*}=\frac{c-2\,\left(1+k\right)\,\left(-1+\delta \right)}{3+2\,k};$$

$$d_{t}^{S^{*}}=\frac{1+k-c\,\left(1+k\right)+2\,\delta +k\,\delta }{3+2\,k};$$

$$d_{s}^{S^{*}}=\frac{1-c-\delta }{3+2\,k};d^{S^{*}}=\frac{2+k-c\,\left(2+k\right)+\delta +k\,\delta }{3+2\,k};$$

$$\pi^{S^{*}}=\frac{\begin{array}{c} c^{2}\,\left(2+k\right)+k\,{\left(1+\delta \right)}^{2}-2\,c\,\left(2+k+\delta +k\,\delta \right)\\ +2\,\left(1+\delta +\delta^{2}\right) \end{array}}{6+4\,k}.$$

According to Proposition 2, we know the firm’s optimal behaviors are affected by the unit product cost *c*, the surplus-value δ, and service capability coefficient *k*. We will further analyze the impacts of these parameters on the firm’s optimal strategies. The results are shown in Lemma 1- Lemma 4.

#### Lemma 1

Under the sharing channel strategy, in the traditional channel, we have:

(i) *The product price* $p_{t}^{S^{*}}$ *is increasing in the unit product cost*, i.e., $\frac{\partial p_{t}^{S^{*}}}{\partial c}=\frac{2+k}{3+2\,k}>0$;
(ii) *The product price* $p_{t}^{S^{*}}$ *is increasing in the surplus-value*, i.e., $\frac{\partial p_{t}^{S^{*}}}{\partial \delta }=\frac{2+k}{3+2\,k}>0$*;*
(iii) *The product price* $p_{t}^{S^{*}}$ *is increasing in the service capability coefficient*, i.e., $\frac{\partial p_{t}^{S^{*}}}{\partial k}=\frac{1-c-\delta }{{\left(3+2\,k\right)}^{2}}>0$.

Lemma 1 shows the impacts of the parameters on the product price in the traditional channel. High unit product cost forces the firm to increase the product price to cover the high unit product cost. High surplus-value means the product is more durable and valuable. The firm can set a higher price for the high-value product. High *k* means introducing the sharing channel will spend the firm more efforts. To cover the efforts of introducing the sharing channel, the firm will raise the sharing price, which will cause some customers with sharing preference to turn to buy and own the product in the traditional channel. With the number of the customers in the traditional channel increases, the firm has an incentive to raise the product price to gain more profit.

#### Lemma 2

Under the sharing channel strategy, in the sharing channel, we have:

(i) *The sharing price* s*is increasing in the unit product cost i.e., $\frac{\partial s^{*}}{\partial c}=\frac{1}{3+2\,k}>0$*;*
(ii) *The sharing price* s*is decreasing in the surplus-value, i.e., $\frac{\partial s^{*}}{\partial \delta }=-\frac{2+2\,k}{3+2\,k}<0$*;*
(iii) *The sharing price* s* *is increasing in the service capability coefficient*, i.e., $\frac{\partial s^{*}}{\partial k}=\frac{2\,\left(1-c-\delta \right)}{{\left(3+2\,k\right)}^{2}}>0$.

According to Lemma 2, we know when the unit product cost is high, the firm will increase the sharing price to cover the high unit product cost. On one hand, high surplus-value means the product is durable. On the other hand, high surplus-value means the loss of the product is low during the process of use. These two reasons cause the firm to decrease the sharing price. High *k* means the firm’s service capacity coefficient is low. Introducing the sharing channel will make the firm put more efforts. To cover the efforts of introducing the sharing channel, the firm will raise the product price in the sharing channel.

#### Lemma 3

In the sharing channel strategy case, we have the sensitivity analysis of market demand as below:

(i) *The market demand of the traditional channel* $d_{t}^{S^{*}}$ *is increasing with the surplus-value*, i.e., $\frac{\partial d_{t}^{S^{*}}}{\partial \delta }=\frac{2+k}{3+2\,k}>0$*;*
(ii) *The market demand of the traditional channel* $d_{t}^{S^{*}}$ *is increasing with the service capability coefficient*, i.e., $\frac{\partial d_{t}^{S^{*}}}{\partial k}=\frac{1-c-\delta }{{\left(3+2\,k\right)}^{2}}>0$*;*
(iii) *The market demand of the sharing channel* $d_{s}^{S^{*}}$ *is decreasing with the surplus-value*, i.e., $\frac{\partial d_{s}^{S^{*}}}{\partial \delta }=-\frac{1}{3+2\,k}<0$*;*
(iv) *The market demand of the sharing channel* $d_{s}^{S^{*}}$ *is increasing with the service capability coefficient*, i.e., $\frac{\partial d_{s}^{S^{*}}}{\partial k}=-\frac{2\,\left(1-c-\delta \right)}{{\left(3+2\,k\right)}^{2}}<0$*;*
(v) *The total market demand* *d*^S*^ *is increasing with the surplus-value*, i.e., $\frac{\partial d^{S^{*}}}{\partial \delta }=\frac{1+k}{3+2\,k}>0$*;*
(vi) *The total market demand* *d*^S*^ *is decreasing with the service capability coefficient*, i.e., $\frac{\partial d^{S^{*}}}{\partial k}=\frac{-1+c+\delta }{{\left(3+2\,k\right)}^{2}}<0$.

**FIGURE 3:**
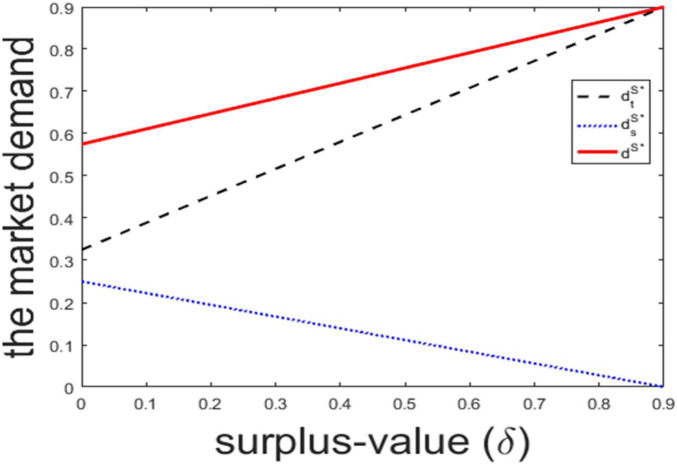
The market demand with surplus-value.

**FIGURE 4:**
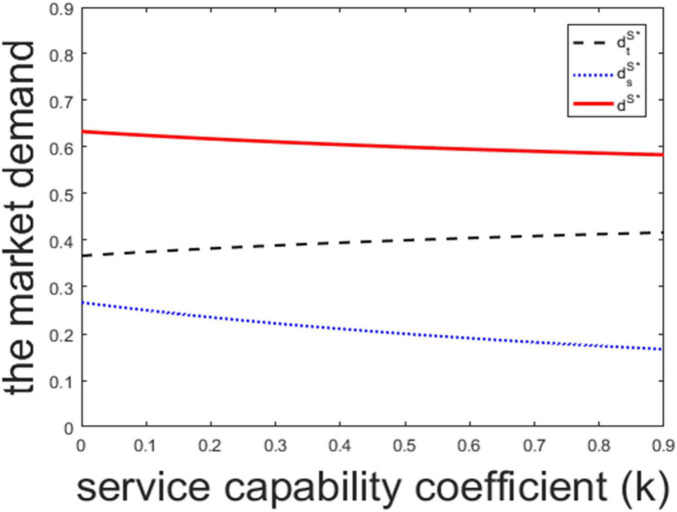
The market demand with service capability coefficient.

According to Lemma 3, we know the market demand in the traditional channel is increasing in the surplus-value, the market demand in the sharing channel is decreasing in the surplus-value, and the total market demand is increasing in the surplus-value. High surplus-value means the product is durable and has a low loss in the process of use. This will improve the customers’ perceived utility, and increase the market demand. With the increasing of perceived utility, customers prefer to purchase and own the product in the traditional channel, which leads the market demand in the traditional channel to increase but the market demand in the sharing channel to decrease. High *k* means the firm’s service capability efficiency is low, and the firm puts more efforts to adopt the sharing channel strategy. To cover the efforts, the firm will raise the product price and sharing price, as stated in Lemma 1 and Lemma 2. This leads to a reduction of the total market demand. Besides, the increased sharing price leads some customers to switch from the sharing channel to the traditional channel. Thus, the market demand in the sharing channel will decrease. There exist two effects in the traditional channel: one is the growth effect caused by the increased sharing price, the other is the diminishing effect caused by the increased price in the traditional channel. The former effect can only partially cover the latter effect, and the market demand in the traditional channel decreases. Figure 3 plots the market demand with surplus-value and Figure 4 plots the market demand with service capability coefficient.

#### Lemma 4

The firm’s total profit when adopting the sharing channel strategy is decreasing in the unit product cost and the service capability coefficient, but increasing in the surplus-value, i.e., $\frac{\partial \pi^{S^{*}}}{\partial c}=\frac{\left(-1+c\right)\,\left(2+k\right)-\left(1+k\right)\,\delta }{3+2\,k}<0$, $\frac{\partial \pi^{S^{*}}}{\partial \delta }=\frac{1+k-c\,\left(1+k\right)+2\,\delta +k\,\delta }{3+2\,k}>0$ and $\frac{\partial \pi^{S^{*}}}{\partial k}=-\frac{{\left(1-c-\delta \right)}^{2}}{2\,{\left(3+2\,k\right)}^{2}}<0$.

As the unit product cost increases, the firm will raise the product price and sharing price to cover the cost. Thus, the product marginal profit will decrease. High product price and high sharing price will decrease customers’ utility both in the traditional channel and in the sharing channel, and cause the market demand to decrease. These two reasons lead to a decrease in the firm’s profit. High surplus-value means the product is durable and the using process is of low loss. Customers prefer to purchase the product and own it. If the sharing price is low, some customers with sharing preferences will turn to the traditional channel. The firm can raise the price of the product with high surplus-value in the traditional channel. There exist two effects: profit growth effect on the traditional channel and profit diminishing effect on the sharing channel. The former effect can completely cover the latter effect and lead the firm’s profit to increase. High *k* means the firm’s service capability efficiency is low, therefore, introducing the sharing channel will make the firm to put more efforts, which will decrease the firm’s profit. Meanwhile, high *k* increases the sharing price, decreases the market demand in the sharing channel, which also leads the firm’s profit reduction. These two reasons cause the firm’s profit to decrease in the firm’s service capability coefficient.

## Results

The comparative analysis results can help us to better understand the impacts of the sharing channel strategy. In this section, we compare the benchmark case and the sharing channel strategy case. The following Proposition 3 shows the impact of the sharing channel strategy on the market demand.

### Proposition 3

Introducing a sharing channel reduces the market demand in the traditional channel, but increases the total market demand, i.e., $d_{t}^{S^{*}}-d_{t}^{B^{*}}<0$, *d*^S*^−*d*^B*^ > 0.

Customers in the market have collaborative consumption behaviors. Adopting the sharing channel strategy is consistent with customers’ collaborative behaviors. This will cause two effects of the market demand. On the one hand, some customers who buy and own the product in the traditional channel turn to the sharing channel, leading to a reduction of the market demand in the traditional channel. On the other hand, the sharing channel strategy provides two alternative distribution channels for customers, which will cause some customers to access the product through the sharing channel. This will cause the growth of the market demand in the sharing channel. Also, the former effect will completely cover the latter effect. These two effects cause the total market demand to expand in the sharing channel strategy case. The relationship of the product price in the traditional channel between these two cases is shown in Proposition 4.

### Proposition 4

The unit product price in the traditional channel under the sharing channel strategy case is lower than the benchmark case, i.e., $p_{t}^{S^{*}}-p_{t}^{B^{*}}<0$.

The sharing channel strategy provides two alternative distribution channels to customers with collaborative consumption behaviors. The sharing channel drives some customers with high perceived usage value for the product to switch to the sharing channel. On the one hand, some customers switching from the traditional channel to the sharing channel reduce the market demand in the traditional channel. As the market demand in the traditional channel decreases, the firm has an incentive to lower the product price. On the other hand, customers with high perceive usage value for the product switch to the sharing channel, making customers remain in the traditional channel have relatively low perceived usage value for the product. This drives the firm to decrease the product price in the traditional channel. We also compare the firm’s profit between these two cases, and the result is shown in Proposition 5.

### Proposition 5

The firm gains more profit by adopting the sharing channel strategy, i.e., π^S*^−π^B*^ > 0.

With the adoption of the sharing channel strategy, the firm can benefit from two aspects. Firstly, the sharing channel strategy helps the firm to expand the total market demand, as stated in Proposition 3. Secondly, the sharing channel strategy better matches customers’ collaborative consumption behaviors and attracts more customers to buy or share the product. Adopting the sharing channel strategy will make the firm put more efforts, but the benefits of the sharing channel strategy are high enough to cover the cost of the efforts. A firm can gain more from adopting the sharing channel strategy.

## Discussion and Conclusion

In the sharing economy, customers have collaborative consumption behaviors. The interaction between customer behaviors and firm decisions has attracted more attention (Yang and Dong, 2017, 2018, etc.). In this paper, we establish an analytical framework to investigate the interaction between customers’ collaborative consumption behaviors and firm’s sharing channel strategy. With adopting the sharing channel strategy, the firm has a chance to expand the market demand and improve its profit.

Firstly, we analyze the benchmark case in which the firm does not adopt the sharing channel strategy. The customers can only buy the product and own it from the traditional channel, therefore, customers’ sharing needs can not be met. Second, we study the sharing channel strategy and characterize the firm’s decisions at the equilibrium. We find the unit product cost, a firm’s service capability coefficient and the surplus-value all play roles in the firm’s adoption of the sharing channel strategy. Third, we make a comparative analysis between the benchmark case and the sharing channel strategy case. The results show that adopting the sharing channel strategy will drive some customers to switch from the traditional channel to the sharing channel, expanding the total market demand and increasing the firm’s profit.

In this paper, we consider a monopoly market. In reality, the market is competitive, a firm needs to react to its competitors’ actions during the decision process. The consideration of the competition effect on the sharing channel strategy could be an interesting extension to this paper. This paper considers that the firm introduces the sharing channel by itself. However, in reality, many firms introduce the sharing channel through the third-party platform. In future research, we will consider channel construction mode as a factor influencing the firm’s sharing channel strategy.

## Data Availability Statement

The original contributions presented in the study are included in the article/Supplementary Material, further inquiries can be directed to the corresponding author/s.

## Author Contributions

SD designed the research and formulated the model. LC provided the solutions and carried out the analysis. ML and ZA modified the writing. All authors contributed to the article and approved the submitted version.

## Conflict of Interest

The authors declare that the research was conducted in the absence of any commercial or financial relationships that could be construed as a potential conflict of interest.

## Publisher’s Note

All claims expressed in this article are solely those of the authors and do not necessarily represent those of their affiliated organizations, or those of the publisher, the editors and the reviewers. Any product that may be evaluated in this article, or claim that may be made by its manufacturer, is not guaranteed or endorsed by the publisher.
