# Supplementary material for: Sharing Channel Strategy With Customers’ Collaborative Consumption Behaviors
Source: Front Psychol. 2022 Mar 28;13:792704. doi: 10.3389/fpsyg.2022.792704 (PMC8996143; doi:10.3389/fpsyg.2022.792704)
Supplement: Supplementary file 1 [file Data_Sheet_1.DOCX]

Supplementary Material

# Supplementary Proof of Propositions and Lemmas

*Proof of Proposition 1*

According to the description of the benchmark case in subsection 4.1, we know the market demand is $d_{t}^{B}=1+\delta-p_{t}^{B}$. So the firm’s profit maximize problem in the benchmark case can be rewritten as below:

$$\mathrm{Max} \pi^{B}=\left( p_{t}^{B}-c \right)(1+\delta-p_{t}^{B})$$

$\pi^{B}$ is a quadratic function of $p_{t}^{B}$. Calculate the second derivative of $\pi^{B}$ with respect to $p_{t}^{B}$, we get $\frac{d^{2}\pi^{B}}{d{p_{t}^{B}}^{2}}=-2<0$. Solve the first-order condition of $\pi^{B}$ on $p_{t}^{B}$, we reach the result $p_{t}^{B}=\frac{1+c+\delta}{2}$. Since $\frac{d\pi^{B}}{dp_{t}^{B}}\left( p_{t}^{B}=c \right)=1-c+\delta>0$ and $\frac{d\pi^{B}}{dp_{t}^{B}}\left( p_{t}^{B}=1 \right)=c+\delta-1<0$, we have the firm’s optimal price in the benchmark case as ${p_{t}^{B}}^{*}=\frac{1+c+\delta}{2}$. Also, we can calculate the optimal market demand and the firm’s optimal profit as ${d_{t}^{B}}^{*}=\frac{1}{2}(1-c+\delta)$ and ${\pi^{B}}^{*}=\frac{1}{4}{(1-c+\delta)}^{2}$, respectively. And, Proposition 1 is proved.

*Proof of Proposition 2*

From the sharing channel strategy description in subsection 4.2, we know the firm’s decision is formulated as below:

$$Max \pi^{S}=\left( p{}_{t}^{S}-c \right)\left( 2\delta+s-p_{t}^{S} \right)+\left( s+\delta-c \right)\left[ 1-\left( \delta+s \right) \right]-\frac{1}{2}k\left[ 1-\left( \delta+s \right) \right]^{2}$$

When $s$ is given, and $\pi^{S}(p_{t}^{S}|s)$ is a quadratic function of $p_{t}^{S}$. By solving the first-order condition of $\pi^{S}(p_{t}^{S}|s)$ on $p_{t}^{S}$, we reach the result $p_{t}^{S}=\frac{1}{2}(c+s+2\delta)$. Since $\frac{d^{2}\pi^{S}\left( p_{t}^{S} | s \right)}{d{p_{t}^{S}}^{2}}=-2<0$, $\frac{d\pi^{S}\left( p_{t}^{S} | s \right)}{dp_{t}^{S}}\left( p_{t}^{S}=c \right)=2\delta+s-c>0$ and $\frac{d\pi^{S}\left( p_{t}^{S} | s \right)}{dp_{t}^{S}}\left( p_{t}^{S}=2\delta+s \right)=c-s-2\delta<0$, we obtain the firm’s optimal price $p_{t}^{S}=\frac{1}{2}(c+s+2\delta)$. Substituting the optimal price $p_{t}^{S}$ into $\pi^{S}(p_{t}^{S}|s)$, we have $\max\pi^{S}\left( s \right)=\frac{1}{4}[c^{2}+2c(-2+s)+4s-3s^{2}+4\delta-4s\delta-2k\left( -1+s+\delta\right)^{2}]$. Since $\frac{d^{2}\pi^{S}\left( s \right)}{ds^{2}}=\frac{1}{4}\left( -6-4k \right)<0$, $\pi^{S}\left( s \right)$ is a concave function of $s$. Solve the first-order condition of $\pi^{S}\left( s \right)$ on $s$, we have $s=\frac{2+c+2k-2\delta-2k\delta}{3+2k}$. Since $\frac{d\pi^{S}\left( s \right)}{ds}\left( s=0 \right)=\frac{c}{2}+\left( 1+k \right)\left( 1-\delta\right)>0$ and $\frac{d\pi^{S}\left( s \right)}{ds}\left( s=1-\delta\right)=-\frac{1}{2}\left( 1-c-\delta\right)<0$, we obtain the firm’s optimal sharing price as $s^{*}=\frac{2+c+2k-2\delta-2k\delta}{3+2k}$. Also, we can calculate the optimal price, the optimal market demand in the traditional channel, the optimal market demand in the sharing channel, the total market demand and the firm’s optimal profit as below:

${p_{t}^{S}}^{*}=\frac{1+k+c \left( 2+k \right)+2 \delta+k \delta}{3+2 k}$; ${d_{t}^{S}}^{*}=\frac{1+k-c(1+k)+2\delta+k\delta}{3+2k}$; ${d_{s}^{S}}^{*}=\frac{1-c-\delta}{3+2k}$; ${d^{S}}^{*}=\frac{2+k-c(2+k)+\delta+k\delta}{3+2k}$; ${\pi^{S}}^{*}=\frac{c^{2}(2+k)+k{(1+\delta)}^{2}-2c(2+k+\delta+k\delta)+2(1+\delta+\delta^{2})}{6+4k}$.

To sum up, Proposition 2 is proved.

*Proof of Lemma 1*

According to Proposition 2, we have:

$\frac{\partial{p_{t}^{S}}^{*}}{\partial c}=\frac{2+k}{3+2k}>0$; $\frac{\partial{p_{t}^{S}}^{*}}{\partial\delta}=\frac{2+k}{3+2k}>0$ and $\frac{\partial{p_{t}^{S}}^{*}}{\partial k}=\frac{1-c-\delta}{{(3+2k)}^{2}}>0$

Lemma 1 is proved.

*Proof of Lemma 2*

According to Proposition 2, we have:

$\frac{\partial s}{\partial c}=\frac{1}{3+2k}>0$; $\frac{\partial s}{\partial\delta}=-\frac{2+2k}{3+2k}<0$ and $\frac{\partial s}{\partial k}=\frac{2(1-c-\delta)}{{(3+2k)}^{2}}>0$

Lemma 2 is proved.

*Proof of Lemma 3*

According to Proposition 2, we have:

$\frac{\partial{d_{t}^{S}}^{*}}{\partial\delta}=\frac{2+k}{3+2k}>0$; $\frac{\partial{d_{t}^{S}}^{*}}{\partial k}=\frac{1-c-\delta}{{(3+2k)}^{2}}>0$; $\frac{\partial{d_{s}^{S}}^{*}}{\partial\delta}=-\frac{1}{3+2k}<0$ ; $\frac{\partial{d_{s}^{S}}^{*}}{\partial k}=-\frac{2\left( 1-c-\delta\right)}{\left( 3+2k \right)^{2}}<0$; $\frac{\partial{d^{S}}^{*}}{\partial\delta}=\frac{1+k}{3+2k}>0$ and $\frac{\partial{d^{S}}^{*}}{\partial k}=\frac{-1+c+\delta}{{(3+2k)}^{2}}<0$

Lemma 3 is proved.

*Proof of Lemma 4*

Similarly, according to Proposition 2, we have:

$\frac{\partial{\pi^{S}}^{*}}{\partial c}=\frac{(-1+c)(2+k)-(1+k)\delta}{3+2k}<0$, $\frac{\partial{\pi^{S}}^{*}}{\partial\delta}=\frac{1+k-c(1+k)+2\delta+k\delta}{3+2k}>0$ and $\frac{\partial{\pi^{S}}^{*}}{\partial k}=-\frac{\left( 1-c-\delta\right)^{2}}{2\left( 3+2k \right)^{2}}<0$

Lemma 4 is proved.

*Proof of Proposition 3*

According to Proposition 1, we know there is only the traditional channel in the benchmark case and the market demand of the traditional channel is equal to the total market demand, i.e., ${d_{t}^{B}}^{*}={d^{B}}^{*}=\frac{1}{2}(1-c+\delta)$.

According to Proposition 2, we know the market demand of the traditional channel and the total market demand are ${d_{t}^{S}}^{*}=\frac{1+k-c(1+k)+2\delta+k\delta}{3+2k}$ and ${d^{S}}^{*}=\frac{2+k-c(2+k)+\delta+k\delta}{3+2k}$, respectively.

By calculating the difference of the market demand in the traditional channel between the benchmark case and the sharing channel strategy case, we get ${d_{t}^{S}}^{*}-{d_{t}^{B}}^{*}=\frac{-1+c+\delta}{6+4k}<0$;

Also, we can calculate the difference of the total market demand between the two cases as ${d^{S}}^{*}-{d^{B}}^{*}=\frac{1-c-\delta}{6+4k}>0$.

Proposition 3 is proved.

*Proof of Proposition 4*

According to Proposition 1 and Proposition 2, we know the unit product prices of the traditional channel in the sharing channel strategy case and in the benchmark case are ${p_{t}^{S}}^{*}=\frac{1+k+c \left( 2+k \right)+2 \delta+k \delta}{3+2 k}$ and ${p_{t}^{B}}^{*}=\frac{1}{2}(1+c+\delta)$, respectively. Solving the difference of the traditional channel price between the two cases, we get ${p_{t}^{S}}^{*}-{p_{t}^{B}}^{*}=\frac{-1+c+\delta}{6+4k}<0$. Proposition 4 is proved.

*Proof of Proposition5*

According to Proposition 1 and Proposition 2, we know the firm’s profit in the sharing channel strategy case and in the benchmark case are ${\pi^{S}}^{*}=\frac{c^{2}(2+k)+k{(1+\delta)}^{2}-2c(2+k+\delta+k\delta)+2(1+\delta+\delta^{2})}{6+4k}$ and ${\pi^{B}}^{*}=\frac{1}{4}{(1-c+\delta)}^{2}$, respectively. Calculating the difference of the firm’s profit between the two cases, we get ${\pi^{S}}^{*}-{\pi^{B}}^{*}=\frac{\left( 1-c-\delta\right)^{2}}{12+8k}>0$. Proposition 5 is proved.
